# Forgiveness, Gratitude, Happiness, and Prosocial Bystander Behavior in Bullying

**DOI:** 10.3389/fpsyg.2019.02827

**Published:** 2020-01-08

**Authors:** Fernanda Inéz García-Vázquez, Angel Alberto Valdés-Cuervo, Belén Martínez-Ferrer, Lizeth Guadalupe Parra-Pérez

**Affiliations:** ^1^Department of Education, Technological Institute of Sonora, Ciudad Obregón, Mexico; ^2^Department of Education and Social Psychology, Pablo de Olavide University, Seville, Spain; ^3^School of Education, Colorado State University, Fort Collins, CO, United States

**Keywords:** forgiveness, gratitude, prosocial behavior, adolescence, happiness

## Abstract

The relationships among character strengths (forgiveness and gratitude), happiness, and prosocial bystander behavior in bullying were analyzed. The sample includes 500 (early adolescents) and 500 (middle adolescents) of both genders, between 12 and 18 years old (*M* age = 14.70, SD = 1.58). Two structural equation models were calculated. Results of the first model indicated that forgiveness, gratitude, and happiness had a direct positive relation with prosocial bystander behavior. Furthermore, human strengths were indirectly related to prosocial behavior in bullying for this effect in happiness. The second model showed that prosocial bystander behavior had a positive effect on human strengths and happiness. Multigroup analyses indicated that gender and stage of adolescence did not moderate the relations found in the model. Overall findings suggest a reciprocal relationship between character strengths, happiness, and prosocial bystander behavior.

## Introduction

Owing to its negative effects on academic and socioemotional development (Vaillancourt et al., 2013; Werth et al., 2015; Graham, 2016), bullying has become an important health issue in school-aged adolescents (Modecki et al., 2014; Davis et al., 2019). Bullying is defined as repetitive and intentionally aggressive behavior against defenseless victims (Olweus, 1993; Camodeca et al., 2002; Volk et al., 2014). Bullying involves not only victims and bullies but also bystanders (Craig et al., 2000; Flaspohler et al., 2009). While bystanders are the most numerous participants in bullying, they are capable of adopting three different behaviors in these events: avoidance, probullying, or prosocial behavior (Salmivalli et al., 1996; Demaray et al., 2016; Alcántar-Nieblas et al., 2018). Therefore, prosocial bystander behavior is essential for maintaining positive relationships, limiting bullying, and for promoting social adjustment in the victims (Salmivalli et al., 2011; Kärnä et al., 2013). A plethora of research has been conducted to further understand the role of victim and aggressor; however, very little is known about the predictors of prosocial behaviors. According to Eisenberg et al. (2015), prosocial behavior is a kind of conduct through which people benefit others. Specifically, it leads bystanders to defend the victims, comfort them, or alert adults about bullying episodes (Sutton and Smith, 1999; Reijntjes et al., 2016). Unsurprisingly, the adoption of prosocial behavior of bystanders contributes to reducing bullying and its negative effects on the victims (Salmivalli, 2010; Polanin et al., 2012; Twemlow and Sacco, 2013; Machackova and Pfetsch, 2016; Jenkins and Nickerson, 2017). Nonetheless, only a small number of bystanders have been found to adopt prosocial behaviors in bullying episodes (Barhight et al., 2013; Olenik-Shemesh et al., 2017; Waasdorp and Bradshaw, 2018). While scholars remain incapable to elucidate the leading factors toward the adoption of prosocial behaviors in bystanders (Barchia and Bussey, 2011; Van der Ploeg et al., 2017; Lambe et al., 2019), they are urged to further explore the underpinnings of prosocial behavior in bullying events.

The first difficulty is finding a research model that explains why individuals adopt prosocial behaviors; this is a complex task due to the different theoretical strands influencing the field (Lapsley and Narvaez, 2013; Garrigan et al., 2018). Some scholars (Ettekal et al., 2015; Lambe et al., 2019) believe that the adoption of prosocial behavior can be explained through moral variables. Bystander researchers often times adopt either cognitive (Kohlberg, 1969; Rest et al., 2000) or affective (Hoffman, 2000; Tangney et al., 2007) frameworks of moral development. Under these perspectives, moral judgment and moral emotions are referred as directly related to the prosocial behavior. For example, some studies analyze the effects of moral sensitivity (Thornberg and Jungert, 2013; Levasseur et al., 2017), self-importance of moral value (Pozzoli et al., 2016), empathy (Pöyhönen et al., 2010; Barchia and Bussey, 2011; Nickerson et al., 2014; Pozzoli et al., 2016; Van der Ploeg et al., 2017; Yun and Graham, 2018), and restorative shame management (Ahmed, 2008; Valdés-Cuervo et al., 2018) with defender bystander behavior in bullying. Nonetheless, for social cognitivist (Blasi, 1980, 1984, 2004; Rest, 1986; Narvaez and Lapsley, 2009; Bergman, 2013), prosocial behavior is a complex phenomenon that cannot be fully explained using either a single or a couple of variables. According to them, neither moral emotions nor moral judgment can fully predict prosocial behavior by themselves; therefore, the exploration of moral identity, over merely morale variables, remains crucial to understanding the underpinnings of prosocial behavior (Blasi, 1980, 1984; Aquino and Reed, 2002; Narvaez and Lapsley, 2009).

According to social cognitivists (Aquino and Reed, 2002; Lapsley and Narvaez, 2013; Miles and Upenieks, 2018), moral identity comprises a set of self-schemas arranged around a group of character strengths. Moral self-schema refers to a stable set of memories that summarize a person’s beliefs, affective experiences, and generalization about the self, in moral domains (Lapsley, 2016). These schemas automatically dispose individuals to seek out and select schema-relevant tasks, goals, and settings that serve to canalize dispositional tendencies (Redd and Aquino, 2003; Dutton et al., 2010; Lapsley, 2016).

### Virtues (Gratitude and Forgiveness)

Virtues refer to the expression of character strengths valued in most cultures (Seligman, 2002; Peterson and Park, 2004). Virtues are moral self-schemas which comprise rational and emotional elements (Emde, 2016). They constitute personality disposition (Emmons, 2008) linked to socially positive behaviors in certain domains (Froh et al., 2009; Donovan and Priester, 2017). In particular, some authors have stressed the relevance of gratitude and forgiveness as interpersonal strengths linked to positive outcomes, including prosocial behaviors (Yamhure-Thompson and Snyder, 2003; Kubacka et al., 2011; Gordon et al., 2012).

The literature mostly refers virtues such as gratitude and forgiveness as moral self-schemas associated with prosocial behavior (Karremans et al., 2005; Yost-Dubrow and Dunham, 2018). Gratitude and forgiveness not only relate to prosocial behavior but also to individuals’ happiness (Strohminger and Nichols, 2014; Phillips et al., 2017; Han et al., 2019). In the past, some scholars (Meier and Stutzer, 2008; Weinstein and Ryan, 2010; Rudd et al., 2014; Light et al., 2015; Sulemana, 2016; Erreygers et al., 2019) have already reported an association between happiness and prosocial behavior. Overall, results from those studies confirm the usefulness not only of the social cognitive theories but also the adoption of a positive psychology approach (Seligman, 2002; Peterson and Seligman, 2004) in understanding the factors that lead individuals to adopt prosocial behaviors.

Gratitude is a virtue that relates to helping behaviors and promotes positive outcomes in individuals (Froh et al., 2010; Jiang et al., 2017; Shiraki and Igarashi, 2018; Yost-Dubrow and Dunham, 2018; Bono et al., 2019). It results from the recognition of others’ good intentions and the appreciation of generous actions as an altruistic gift (Lazarus and Lazarus, 1994; Emmons, 2009). In line with this concept, other authors have defined gratitude as a positive assessment of the benefits contributed by others to personal experiences (McCullough et al., 2002, 2008; Froh et al., 2010). As expected, some scholars have reported finding that adolescents with higher levels of gratitude have a positive perception of others and are prone to adopt prosocial behaviors (Rey and Extremera, 2014; Foster et al., 2017; Reckart et al., 2017). Prior studies have also shown that gratitude has a positive impact on adolescent happiness, as it involves reflection, positive emotions, adaptive social behaviors, and relationships that facilitate well-being (Fredrickson, 2004; Watkins, 2004; Armenta et al., 2017). In fact, it has been reported that people with higher levels of gratitude experience less anger, feelings of loneliness, and fewer depressive symptoms (Breen et al., 2010; Wu et al., 2018; Rey et al., 2019).

On the other hand, forgiveness involves both a benevolent feeling toward the offender and the re-establishment of the relationship in terms of trust and hope (Ahmed and Braithwaite, 2006). This strength concerns a set of coping responses that encompass an increase in positive attitude and prosocial approach to others (Worthington, 1998; Hui and Ho, 2004; Flanagan et al., 2012). Forgiveness has been related to the engagement in positive strategies focused on solving interpersonal conflicts in adolescents (Ahmed and Braithwaite, 2006; Egan and Todorov, 2009; Flanagan et al., 2012; Quintana-Orts and Rey, 2018; León-Moreno et al., 2019) and prosocial behaviors (Karremans et al., 2005; Karremans and Van Lange, 2008; Riek and DeWit, 2018). Moreover, forgiveness helps adolescents to maintain close bonds and increase their happiness (McCullough, 2000; Maltby et al., 2005).

While recent studies show that gratitude and forgiveness have the twofold benefit of decreasing the risk of aggression and victimization and also reducing the negative consequences of being bullied (Karremans et al., 2005; Egan and Todorov, 2009; Riek and DeWit, 2018; Rey et al., 2019; Yu and Chan, 2019), little is known about their relations with bystander behavior.

### Happiness

Happiness is the result of positive affective and cognitive evaluation of life (Diener, 2000; Veenhoven, 2010). The literature mostly associates happiness with pleasure (seeking gratifications and avoidance of discomfort), commitment (involvement in goal-related activities important for the individual), and meaning (use of personal virtues and skills for a greater good) (Seligman, 2002; Peterson et al., 2005; Park et al., 2009). Some scholars suggest that happiness is associated with character strengths (Park and Peterson, 2006; Proctor et al., 2010; Mongrain et al., 2011; Rana et al., 2014). Moreover, happiness plays an important role in adolescent positive outcomes (Park and Peterson, 2006; Proctor et al., 2010; Mongrain et al., 2011; Rana et al., 2014). In fact, evidence suggests that those who are happier are more likely to exhibit prosocial behavior in the future (Thoits and Hewitt, 2001; Walker, 2007; Meier and Stutzer, 2008; Rudd et al., 2014; Light et al., 2015; Sulemana, 2016). However, no studies were found reporting on the relationship between happiness and prosocial bystander behavior in bullying.

### The Present Research

Most of the studies exploring prosocial bystander behavior in bullying use theoretical frameworks around moral judgment or emotions. Despite the larger evidence suggesting that character strengths and happiness are associated with prosocial behavior (Light et al., 2015; Riek and DeWit, 2018; Yost-Dubrow and Dunham, 2018), no study known by the authors has explored the relationship between forgiveness, gratitude, happiness, and prosocial behavior in bullying. Furthermore, the literature regarding bystanders’ behavior in bullying events within the Mexican context is still scarce.

**FIGURE 1:**
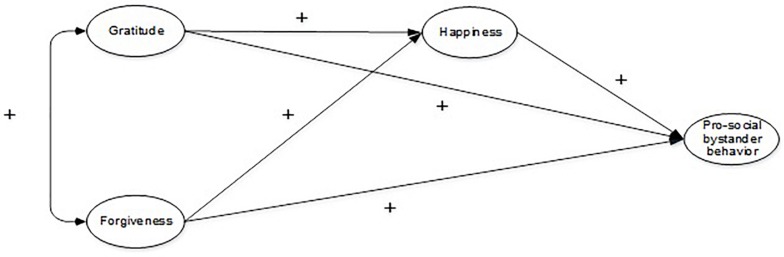
Theoretical model of the relations between forgiveness, gratitude, happiness, and prosocial bystander behavior.

As other scholars (Blasi, 1980, 1984; Aquino and Reed, 2002; Narvaez and Lapsley, 2009), the authors posit that moral identity is crucial to explain moral behavior. Therefore, unlike previous studies, we adopted a social-cognitive model of moral behavior (Hardy, 2006; Aquino et al., 2009; Narvaez and Lapsley, 2009; Lapsley and Narvaez, 2013). Within this model, individual identity is seen as a self-schema, which is organized around the association of specific moral traits. Moreover, based on the current body of literature, the mediation effect of happiness was analyzed with the association between forgiveness, gratitude, and bystander prosocial behavior. Specifically, this study explored the direct and indirect relationships between gratitude, forgiveness, happiness, and prosocial bystander behavior in Mexican early and middle adolescents (see Figure 1). Moreover, an alternative model was used to explore the effect of prosocial behavior on human strengths and happiness, since reciprocal relations between these positive factors are understudied. Finally, the role of gender and stage of adolescence (early vs. middle) in these relations was also examined.

Since previous scholars have reported direct and indirect positive relations between gratitude, forgiveness, happiness, and prosocial behavior (Meier and Stutzer, 2008; Rudd et al., 2014; Light et al., 2015; Sulemana, 2016; Riek and DeWit, 2018; Yost-Dubrow and Dunham, 2018), a positive relationship between these variables and prosocial behavior in bullying was anticipated. Moreover, prosocial behavior was expected to influence character strengths and happiness, as previous empirical evidence has shown (Lerner et al., 2003; Dunn et al., 2014; Aknin et al., 2015; Carlo et al., 2015; Fu et al., 2017; Al-yaaribi et al., 2018; Bieda et al., 2019). Finally, no hypothesis was made about the effect of gender and adolescence stage in the proposed model due to the fact that the current literature is inconclusive and even contradictory (Carlo et al., 2007, 2015; Luengo Kanacri et al., 2013; Flynn et al., 2014; Van der Graaff et al., 2014; Padilla-Walker et al., 2018).

## Materials and Methods

### Participants

There were 500 early and 500 middle adolescents from 28 public schools in the state of Sonora, Mexico. Participants were selected by simple probabilistic sampling (*p* = 0.5, *q* = 95%). The sample of early adolescents included 215 (45%) female (*M* age = 12.35, SD = 0.81 years) and 286 (55%) male (*M* age = 12.37, SD = 0.73 years) ranging from 12 to 15 years old. The middle adolescent sample included 262 (52.5%) female (*M* age = 17.11, SD = 0.94 years) and 237 (47.5%) male (*M* age = 16.97, SD = 0.94 years) with ages between 16 and 18 years old. As most public urban institutions in Mexico, its population includes students from low and middle classes (National Institute for the Evaluation of Education [INEE], 2018).

### Measures

#### Gratitude

*The Gratitude Questionnaire* (GQ6; McCullough et al., 2002) was used. According to McCullough et al. (2008), gratitude is a positive assessment of the benefits that others brought to personal experiences. This is a one-dimension scale, which consists of four items (e.g., *If I had to make a list of things for which I am grateful, it would be a long list*, α = 0.88, Ω = 0.90). The scale responses are in Likert-type format with seven options (0 = *strongly disagree*, 6 = *strongly agree*). The confirmatory factor analysis (CFA) showed a good fit of the model [*X*^2^ = 2.39, df = 1, *p* = 0.122; standardized root-mean-square residual (SRMR) = 0.01; adjusted goodness-of-fit index (AGFI) = 0.98; comparative fit index (CFI) = 0.99; root mean square error of approximation (RMSEA) = 0.03, 90% CI (0.00, 0.10)].

#### Forgiveness

The *Forgiveness Heartland Scale* (Thompson et al., 2005) was used. The authors define forgiveness as an action that involves both a benevolent feeling toward the offender and the re-establishment of the relationship. This one-dimension scale consists of eight (e.g., *Eventually I understand the mistakes that others make*, α = 0.75, Ω = 0.81) Likert-type items (0 = *strongly disagree*, 6 = *strongly agree*). The CFA showed a good fit of the measurement model [*X*^2^ = 33.07, df = 10, *p* = 0.337; SRMR = 0.04; AGFI = 0.99; CFI = 0.99; RMSEA = 0.04, 90% CI (0.02, 0.05)].

#### Happiness

The scale of *Orientation to Happiness* (Peterson et al., 2005) was used. This scale consists of 10 Likert-type items (0 = *completely opposite to me*, 4 = *Very much like me*, α = 0.82), referring to three orientations to happiness: pleasure that seeks for fulfillment and avoidance of discomfort (e.g., *The good life is full of pleasure*); commitment, defined as the involvement in goal-related activities important for the individual (e.g., *When I have to choose what to do, I always take into account if I can commit*); and life with meaning, the use of personal virtues and skills for a greater good (e.g., *my life has a bigger purpose*). The CFA showed a good fit of the unidimensional model [*X*^2^ = 21.93, df = 14, *p* < 0.08; SRMR = 0.03; AGFI = 0.98; CFI = 0.99; RMSEA = 0.02, 90% CI (0.01, 0.04)].

#### Prosocial Bystander

To assess the prosocial bystander role, a subscale from the participant role approach was used (Alcántar-Nieblas et al., 2018). Prosocial behavior refers to the adolescents’ involvement in behaviors aimed at protecting or comforting the victims of bullying (e.g., when a classmate is physically assaulted I inform the adults, α = 0.80, Ω = 0.82). The subscale consists of four Likert-type items (0 = *never*, 4 = *always*). The participant role approach was found valid in Mexican populations [*X*^2^ = 95.41, df = 60, *p* = 0.002; SRMR = 0.05; AGFI = 0.95; CFI = 0.96; RMSEA = 0.03, 90% CI (0.01, 0.04)].

### Procedure

The study was approved by the Ethics Committee of the Technological Institute of Sonora. Then, principals and teachers from schools located at Sonora, Mexico were reached to gather voluntary participants. Later, a consent letter was sent to the adolescents’ parents both explaining the purpose of the study and asking permission for students’ participation. Only 3% of parents refused to allow their children to participate in the study. Finally, we ensured voluntary participation and the confidentiality of their participation. The participants were informed that they may leave the study at any time during the data collection process.

### Data Analysis

The percentage of missing data was 4% in the sample. In all cases, they were treated using the SPSS imputation method. During the first stage, both descriptive (median and deviation standard) and univariate analysis (bivariate correlation, media comparison, and size of effect) were conducted. Later, CFA, the invariance measurement of scales, structural equation model, and structural invariance, using AMOS software were also used. All the analysis used the maximum likelihood estimation with percentile method bootstrap (with 5,000 replicates and a 95% confidence interval) to determine the goodness of fit of the models. The bootstrap is an AMOS method to approach multivariate normality issues (Hancock and Liu, 2012; Byrne, 2016).

#### Measurement Invariance

Multigroup analyses (Millsap and Olivera-Aguilar, 2012; Byrne, 2016; Schumacker and Lomax, 2016) were utilized by testing gender and stage of adolescence invariance in the measurements. In the analysis of invariance, configurational invariance (baseline model), metric invariance (factor loading), and scalar (measurement intercept) were evaluated. The measurement and structural invariance of the model between early and later adolescents were made using indicators of invariance Δ*X*^2^ with *p* > 0.001, ΔCFI < 0.01, and ΔRMSEA < 0.015 (Sass and Schmitt, 2013; Byrne, 2016).

#### Structural Model

The goodness of fit of the structural models were calculated using chi-squared and associated probability (*X*^2^ with *p* > 0.001), SRMR (≤0.08), Tucker–Lewis index (TLI ≥ 0.95), AGFI (≥0.95), CFI (≥0.95), and RMSEA (≤0.05) (Blunch, 2013; Byrne, 2016; Kline, 2016). The direct and indirect effects were calculated with the bootstrap percentile method. For the comparison of alternative models, the Akaike Information Criterion (AIC) and Bayesian information criterion (BIC) were used (Blunch, 2013; Byrne, 2016; Kline, 2016).

#### Structural Invariance

The structural coefficients invariance was tested concerning the relationships among the variables included in the model (gratitude, forgiveness, happiness, and prosocial bystander behavior). To test invariance of structural coefficients, five paths were constrained equal over gender and adolescence stage (early and middle). The following recommendations were applied for multigroup invariance testing: Δ*X*^2^ with *p* > 0.001, ΔCFI < 0.01, and ΔRMSEA < 0.015 (Sass and Schmitt, 2013; Byrne, 2016).

## Results

### Descriptive and Univariate Analysis

The results showed a significant positive correlation between gratitude, forgiveness, happiness, and prosocial behavior in bullying. Moreover, female participants scored higher than male participants in all the analyzed variables, and early adolescents scored higher in happiness than middle adolescents; no other differences in both stages were found (see Table 1).

**TABLE 1:** Means, standard deviations, correlations, and mean comparisons by gender and stage of adolescence.

**Variable**	***M***	**SD**	**1**	**2**	**3**	**4**
(1) Prosocial bystander	1.84	1.08	−			
(2) Gratitude	4.69	1.39	0.20^∗∗∗^	−		
(3) Forgiveness	3.78	1.31	0.18^∗∗∗^	0.57^∗∗∗^	−	
(4) Happiness	2.29	0.90	0.29^∗∗∗^	0.49^∗∗∗^	0.49^∗∗∗^	−
***M/*SD**						
Male			1.72/1.01	4.45/1.50	3.62/1.38	2.19/0.87
Female			1.94/1.14	4.89/1.26	3.91/1.25	2.37/0.91
Student *t*			−3.32^∗∗^	−5.01^∗∗∗^	−3.47^∗∗^	−3.12^∗^
Cohen *d*			0.20	0.32	0.22	0.20
***M/*SD**						
Early adolescent			1.81/1.07	4.74/1.35	3.85/1.30	2.34/0.88
Middle adolescent			1.88/1.10	4.64/1.63	3.70/1.33	2.23/0.92
Student *t*			−1.02	1.14	1.87	1.94^∗^
Cohen *d*			0.06	0.07	0.11	0.12

*^∗^*p* < 0.05, ^∗∗^*p* < 0.01, and ^∗∗∗^*p* < 0.001.*

### Measurement Invariance

The measurement invariance over gender (477 female vs. 533 male) and adolescence stage (early 501 vs. middle 509) were tested by examining the following models of invariance: configurational, metric, and scalar model of invariance. Overall, the results showed the invariance of all the measurements used (see Tables 2, 3). Thus, these finding supported the invariance of the different instruments for gender and stage of adolescence.

**TABLE 2:** Test of gender invariance: summary of goodness of-fit statistics.

**Measurement**	**Invariance model**	***X*^2^**	**df**	**Δ*X*^2^**	**Δdf**	***p***	**ΔCFI**	**ΔRMSEA**
Gratitude	Configurational	6.28	2			0.043		
	Metric	9.04	4	2.75	2	0.253	< 0.000	0.01
	Scalar	19.53	5	13.26	3	0.004	0.005	< 0.000
Forgiveness	Configurational	86.61	34			0.000		
	Metric	89.95	41	3.33	7	0.852	0.002	< 0.000
	Scalar	95.45	42	8.84	8	0.356	< 0.000	< 0.003
Happiness	Configurational	32.34	28			0.261		
	Metric	36.85	34	4.50	6	0.609	0.002	0.001
	Scalar	37.40	35	5.05	7	0.653	0.002	0.003
Bystander prosocial behavior	Configurational	1.19	2	0.550				
	Metric	3.58	4	2.38	2	0.303	0.000	0.001
	Scalar	7.36	5	6.17	3	0.104	0.005	0.002

**TABLE 3:** Test of stage of adolescence invariance: summary of goodness-of-fit statistics.

**Measurement**	**Invariance model**	***X*^2^**	**df**	**Δ*X*^2^**	**Δdf**	***P***	**ΔCFI**	**ΔRMSEA**
Gratitude	Configurational	2.37	2			0.305		
	Metric	6.19	5	3.82	3	0.281	0.001	< 0.000
	Scalar	9.35	6	6.98	4	0.137	0.002	0.001
Forgiveness	Configurational	86.56	34			0.000		
	Metric	95.53	41	8.96	7	0.255	0.001	< 0.000
	Scalar	95.61	42	9.04	8	0.338	0.001	< 0.000
Happiness	Configurational	35.38	28			0.159		
	Metric	42.30	34	6.92	6	0.328	0.001	< 0.00
	Scalar	46.45	35	11.07	7	0.135	0.004	< 0.00
Bystander prosocial behavior	Configurational	3.89	2			0.143		
	Metric	4.77	5	0.88	3	0.830	0.001	0.003
	Scalar	5.42	6	1.53	4	0.821	0.001	0.003

### Structural Model

**FIGURE 2:**
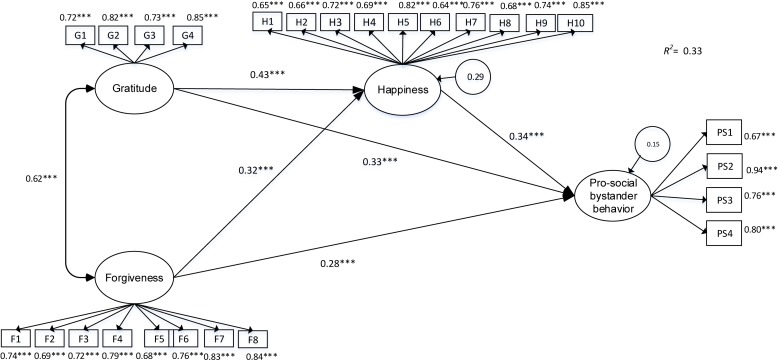
Results of the structural model of the relations between forgiveness, gratitude, happiness, and prosocial bystander behavior. Standardized coefficients are presented.

The calculated structural model showed good fit indices [*X*^2^ = 172.16, df = 126, *p* = 0.04, TLI = 0.98; SRMR = 0.05; AGFI = 0.97; CFI = 0.99; RMSEA = 0.03, 95% CI (0.02, 0.05); AIC = 230.64; BIC = 441.67], explaining 35% of variance in prosocial bystander behavior. Figure 2 shows the structural model with the standardized coefficients and their associated probability. Regarding the direct effects, the results showed that gratitude (β = 0.43, *p* < 0.000) and forgiveness (β = 0.32, *p* < 0.000) have a positive relationship with happiness and prosocial bystander behavior (β = 0.33, *p* < 0.000; β = 0.27, *p* < 0.000). Moreover, the indirect effects showed that gratitude [β = 0.12, *p* < 0.000, 95% CI (0.04, 0.13)] and forgiveness [β = 0.08, *p* < 0.000, 95% CI (0.03, 0.10)] were related to prosocial bystander behavior through happiness.

### Alternative Model

The effects of prosocial bystander behavior in happiness, forgiveness, and gratitude were analyzed. The structural model had an acceptable fit index [*X*^2^ = 218.57, df = 129, *p* < 0.000; SRMR = 0.06; AGFI = 0.94; TLI = 0.93; CFI = 0.95; RMSEA = 0.06, 90% CI (0.04, 0.04), AIC = 260.64; BIC = 470.67]. Prosocial behavior was directly related to happiness (β = 0.34, *p* < 0.000) and gratitude (β = 0.34, *p* < 0.000); however, it was not related to forgiveness (β = 0.02, *p* = 0.62). Regarding indirect effects, prosocial behavior was found to promote forgiveness [β = 0.27, *p* < 0.000, 95% CI (0.18, 0.36)] and gratitude [β = 0.10, *p* < 0.000, 95% CI (0.04, 0.13)] through its positive relationship with happiness. However, this model resulted in a poorer fit than the original model.

### Multigroup Structural Analysis

To analyze the moderated effect of gender and stage of adolescence (early vs. middle), a multigroup analysis was performed. Results showed the existence of structural invariance in gender [*X*^2^ = 376.25, df = 160, *p* < 0.000; CFI = 0.96; AGFI = 0.95; TLI = 0.97; RMSEA = 0.04, 95% CI 90 (0.02, 0.06)] and stage of adolescence [*X*^2^ = 389.36, df = 183, *p* < 0.00; CFI = 0.96; AGFI = 0.93; TLI = 0.94; RMSEA = 0.05, 95% CI (0.03, 0.07)]. Both differences in chi-squared (Δ*X*^2^), in the comparative goodness-of-fit indices (ΔCFI), and RMSEA (ΔRMSEA) suggest that gender and the adolescence stage (early vs. middle) does not affect the relations proposed in the model (see Table 4).

**TABLE 4:** Results of the invariance of structural model by gender and stage of adolescence.

**Gender**	***X*^2^**	**df**	**Δ*X*^2^**	**Δdf**	***p***	**ΔCFI**	**ΔRMSEA**
Configurational	376.25	160					
Metric	385.62	170	9.37	10	0.497	0.001	0.04
Structural weight	389.59	177	13.34	17	0.713	0.003	0.06
Structural covariates	409.65	183	33.39	23	0.74	0.002	0.07
Residual weight	420.74	185	44.48	25	0.10	0.003	0.09

**Adolescents’ stage**	***X*^2^**	**df**	**Δ*X*^2^**	**Δdf**	***p***	**ΔCFI**	**ΔRMSEA**

Configurational	389.36	183					
Metric	370.01	160	9.23	10	0.510	0.000	0.02
Structural weight	379.25	170	14.40	17	0.638	0.000	0.03
Structural covariates	384.42	177	19.34	23	0.681	0.000	0.05
Residual weight	389.36	183	22.71	25	0.594	0.000	0.06

## Discussion

While past studies have focused on the moral domain, they have been unable to fully explain the leading factors for prosocial behavior in bystanders of bullying. Unlike past studies, this research was framed by the social-cognitive model of moral behavior (Aquino and Reed, 2002; Lapsley and Narvaez, 2013; Miles and Upenieks, 2018) and analyzed the association between virtues (forgiveness, gratitude, and happiness) and prosocial bystander behavior. The results showed positive relations between forgiveness, gratitude, happiness, and prosocial bystander behavior. Finally, results indicated that the relationships between these variables were not moderated by gender or adolescence stage (early vs. middle).

Consistent with previous studies, forgiveness and gratitude were related to happiness in adolescents (Maltby et al., 2005; Rana et al., 2014; Witvliet et al., 2018; You et al., 2018). This association may be explained by the influence of forgiveness and gratitude in maintaining positive relationships, which contributes to gaining social support (Hobfoll, 1989; Fredickson, 1998; You et al., 2018). Similar to other studies (Karremans et al., 2005; Meier and Stutzer, 2008; Gini et al., 2011; Seider et al., 2013; Light et al., 2015; Sulemana, 2016; Shiraki and Igarashi, 2018; Bono et al., 2019), these results showed a positive relation between both moral self-schemes and prosocial bystander behavior. In this regard, as other scholars (McCullough et al., 2002; Emmons, 2009; Karremans and van Lange, 2009), we posit gratitude and forgiveness usually evolve to stimulate reciprocity and sympathy, not only with benefactors but also unrelated others. Finally, in line with previous research (Thoits and Hewitt, 2001; Walker, 2007; Meier and Stutzer, 2008; Rudd et al., 2014; Light et al., 2015; Sulemana, 2016), these results indicated that the happiest people are likely to engage in prosocial behaviors. In this regard, some scholars (Thoits and Hewitt, 2001; Dunn et al., 2008; Aknin et al., 2012) explain that happiness produces generosity to others that will be later transformed into prosocial behavior. Overall, the findings suggest individual virtues as a moral self-schema plays an important role in explaining prosocial bystander behavior in bullying.

Results from the alternative model suggest that prosocial bystander behavior positively relates to virtues and happiness in adolescence. This evidence is consistent with past literature that reports a positive impact of prosocial behavior in virtues development (Lerner et al., 2003; Dunn et al., 2014; Aknin et al., 2015; Carlo et al., 2015; Fu et al., 2017; Al-yaaribi et al., 2018; Curry et al., 2018; Bieda et al., 2019). Although the mechanism underlying this relationship remains unclear, the current body of the literature suggests that the effect of happiness in prosocial behavior is related to adolescents’ perceptions about positive consequences of their behavior in their own and others’ well-being (Bierhoff, 2002; Rudd et al., 2014; Paulus and Moore, 2016).

Finally, the gender and adolescence stage did not have a moderating effect on the relationships in the study model. In other words, the influence of gratitude and forgiveness on prosocial bystander behavior in bullying was similar in both genders and adolescence stages. This suggests that forgiveness, gratitude, and happiness are valuable, regardless of gender or stage of adolescence.

### Limitations

The present study contributes to advance the understanding of the relationships between forgiveness, gratitude, happiness, and prosocial bystander behavior. Nonetheless, this study had at least three limitations. First, a cross-sectional design does not allow for probing a causal relationship among the proposed variables. Therefore, longitudinal or experimental designs are suggested. Second, the measures of variables were self-reported, paper-and-pencil measures. Although the findings were consistent to prior research, studies using multiple methods of measure are desirable. Third, despite the relatively large sample, it is necessary to use research samples with greater diversity to generalize the findings.

### Theoretical and Practical Implications

From a theoretical perspective, these findings confirmed the value of the social-cognitive model of moral behavior (Bandura, 1986; Aquino and Reed, 2002; Lapsley and Narvaez, 2013; Miles and Upenieks, 2018) in the understanding on moral behavior. Particularly, results suggest that the activation of moral self-schemas in bullying bystanders predict their moral behavior. Moreover, the evidence shows that giving to students the opportunity to practice prosocial behaviors toward bullying victims favors the accessibility to moral self-schemas in future events. In accordance with the proposed model, the results suggest that individual behavior is consistent with a moral self-schema, which is also related to individuals’ well-being. In summary, results confirm that the activation of moral-self schemas such as gratitude and forgiveness provide bystanders a dispositional readiness to selecting situationally appropriate behavior in the context of bullying. Hence, the exploration of moral self-schemas, particularly virtues, remain a promising field of study in the context of bullying.

From a practical perspective, these findings suggest that promoting virtues along with happiness results in a useful strategy to promote prosocial behavior toward bullying victims and potentially reduce bullying in general. Therefore, we believe that further efforts should target the development of virtues such as gratitude and forgiveness rather than highlighting adolescents’ weaknesses. Hence, we believe it is necessary to link the community, families, and schools to promote a social climate that facilitates the development of these virtues in adolescents. Furthermore, adults should develop the mechanisms to encourage adolescents to engage in prosocial behaviors such as volunteering, charity, community service, etc. Overall, we believe that a caring and righteous environment remain a fundamental source for the development of positive outcomes in adolescents.

## Data Availability Statement

The datasets generated for this study are available on request to the corresponding authors.

## Ethics Statement

The studies involving human participants were reviewed and approved by Bioethics Research Committee from the Technological Institute of Sonora. Written informed consent to participate in this study was provided by the participants’ legal guardian/next of kin.

## Author Contributions

FG-V designed the research methodology, participated in the data analysis stage, and wrote some sections of the manuscript. AV-C participated in the data analysis and wrote some sections of the manuscript. BM-F and LP-P participated in writing some sections of the manuscript.

## Conflict of Interest

The authors declare that the research was conducted in the absence of any commercial or financial relationships that could be construed as a potential conflict of interest.
